# The impact of volume loading–induced low pressure baroreflex activation on arterial baroreflex‐controlled sympathetic arterial pressure regulation in normal rats

**DOI:** 10.14814/phy2.13887

**Published:** 2018-10-11

**Authors:** Yasuhiro Oga, Keita Saku, Takuya Nishikawa, Takuya Kishi, Tomoyuki Tobushi, Kazuya Hosokawa, Takeshi Tohyama, Takafumi Sakamoto, Kenji Sunagawa, Hiroyuki Tsutsui

**Affiliations:** ^1^ Department of Cardiovascular Medicine Kyushu University Graduate School of Medical Sciences Fukuoka Japan; ^2^ Department of Advanced Risk Stratification for Cardiovascular Diseases Center for Disruptive Cardiovascular Medicine Kyushu University Fukuoka Japan; ^3^ Department of Therapeutic Regulation of Cardiovascular Homeostasis Center for Disruptive Cardiovascular Medicine Kyushu University Fukuoka Japan

**Keywords:** Arterial baroreflex, circulatory homeostasis, low pressure baroreflex, sympathetic nerve activity

## Abstract

Although low pressure baroreflex (LPB) has been shown to elicit various cardiovascular responses, its impact on sympathetic nerve activity (SNA) and arterial baroreflex (ABR) function has not been fully elucidated. The aim of this study was to clarify how volume loading‐induced acute LPB activation impacts on SNA and ABR function in normal rats. In 20 anesthetized Sprague‐Dawley rats, we isolated bilateral carotid sinuses, controlled carotid sinus pressure (CSP), and measured central venous pressure (CVP), splanchnic SNA, and arterial pressure (AP). We infused blood stepwise (3 mL/kg/step) to activate volume loading‐induced LPB. Under the ABR open‐loop condition, stepwise volume loading markedly increased SNA by 76.8 ± 21.6% at CVP of 3.6 ± 0.2 mmHg. In contrast, further volume loading suppressed SNA toward the baseline condition. Bilateral vagotomy totally abolished the changes in SNA by volume loading. To assess the impact of LPB on ABR function, we changed CSP stepwise. Low volume loading (CVP = 3.6 ± 0.4 mmHg) significantly shifted the sigmoidal CSP–SNA relationship (central arc) upward from baseline, whereas high volume loading (CVP = 5.4 ± 0.4 mmHg) returned it to the baseline level. Volume loading shifted the linear SNA–AP relationship (peripheral arc) upward without significant changes in slope. In conclusions, volume loading‐induced acute LPB activation evoked two‐phase changes, an initial increase followed by decline from baseline value, in SNA via resetting of the ABR central arc. LPB may contribute greatly to stabilize AP in response to volume status.

## Introduction

The sympathetic nervous system has been shown to play a pivotal role in circulatory homeostasis. Various receptors sense vital variables such as blood pressure, oxygen saturation, and pH and transmit to the brainstem through afferent nerves, and in turn modulate efferent sympathetic nerve activity (SNA) (Guyenet 2006; Floras 2009). Since SNA strongly contributes to both dynamic and static arterial pressure (AP) regulation by changing heart rate (HR), cardiac contractility, arterial resistance and stressed blood volume (Sakamoto et al. 2015), the failure of SNA regulation destabilizes AP and leads to poor outcome in various cardiovascular diseases (Barron and Viskin 1998; Shan et al. 2001; Eguchi et al. 2012). Thus, to clarify the mechanism and the extent by which each particular reflex modifies SNA is important to understand the physiological role of the reflexes and the mechanism of circulatory homeostasis.

The existence of low pressure baroreceptors in the central vein, pulmonary vessels, and heart has been documented and recognized as an important factor of circulatory homeostasis (Coleridge and Kidd 1960; Coleridge et al. 1961). In 1915, Bainbridge showed increases in venous pressure induced tachycardia in dogs, and named this effect “the Bainbridge reflex” Bainbridge 1915). More recently, an atrial distension was shown to activate the Bainbridge reflex and increase efferent cardiac SNA (Bergström et al. 1971). In contrast, numerous studies have shown that receptors embedded in the cardio‐pulmonary region sensed blood accumulation and inhibited renal SNA via vagal afferent nerves (Badoer et al. 1998; Morita and Vatner 1985; Pyner et al. 2002; Ricksten and Thoren 1980). In addition, Bezold and Jarisch (Bell et al. 1993; Mark 1983) verified that cardiac ischemia and chemical agents such as serotonin agonists and bradykinin induced bradycardia and hypotension through the inhibition of SNA (Bezold‐Jarisch reflex). Taken together, those acute alteration of SNA by the low pressure baroreflex (LPB) varies among experimental conditions such as how LPB was activated, where SNA was recorded.

Furthermore, the arterial baroreflex (ABR) is the major determinant of SNA. Since ABR operates as a negative feedback system and mainly regulates SNA to stabilize AP, the closed‐loop operation of ABR obscures the effects of other reflex, including LPB, on SNA and AP. The ABR system can be opened by separating into two subsystems; the central arc that governs how the baroreceptor pressure changes SNA, and the peripheral arc that determines how the SNA changes AP (Sato et al. 1999). Thus, the ABR open loop analysis enables us to understand the pure effects of LPB on SNA and ABR sympathetic AP regulation.

In this study, we defined the LPB as the acute SNA response elicited by systemic volume infusion under ABR open loop condition. The purpose of this investigation was to clarify how volume loading‐induced LPB activation impacts on SNA and ABR function in normal rats.

## Materials and Methods

### Animals and surgical preparations

Experiments and animal care were approved by the Committee on Ethics of Animal Experiment, Kyushu University Graduate School of Medical Sciences, and performed in strict accordance with the Guide for the Care and Use of Laboratory Animals published by the US National Institutes of Health.

The rats used in this study were purchased from Japan SLC, Inc. (Hamamatsu, Japan). During the entire experiment, all the rats were housed in a room maintained at constant temperature (25 ± 2°C) in a 12 h light/dark cycle and fed a normal diet (CE‐2, Nihon CLEA, Tokyo, Japan) and water ad libitum. We used 12–16‐week‐old Sprague–Dawley rats (*n* = 40; body weight = 470 ± 24 g). Of the 40 rats, 20 were for volume‐loading experiments and the remaining 20 for blood collection to be used in volume infusion. We induced anesthesia by an intraperitoneal injection (2 mL/kg) of a mixture of urethane (Sigma‐Aldrich Japan G.K., Tokyo, Japan; 250 mg/mL) and *α*‐chloralose (Sigma‐Aldrich; 40 mg/mL), and maintained the depth of anesthesia with intravenous infusion (2–3 mL/kg/h) of 20‐fold diluted anesthetic mixture from the right jugular vein. We maintained body temperature at approximately 37°C using a heating pad. We mechanically ventilated each animal with oxygen‐enriched gas. To record AP, a fluid‐filled catheter was inserted into the right femoral artery. We also measured central venous pressure (CVP) by inserting a fluid‐filled catheter into the right jugular vein. A thoracotomy sufficiently large to eliminate the effect of intrathoracic pressure was performed. We exposed a postganglionic branch of the splanchnic sympathetic nerve through a right flank incision and attached a pair of stainless steel wire electrodes (Bioflex Wire AS632; Cooner Wire, CA). We secured and insulated the nerve and electrodes with silicone glue (Kwik‐Sil; World Precision Instruments, FL). To quantify SNA, preamplified nerve signal was band‐pass filtered at 150–1000 Hz and full‐wave rectified and low‐pass filtered with a cutoff frequency of 30 Hz using analog circuits as described previously (Saku et al. 2014, 2017). Continuous infusion of pancuronium bromide (MIOBLOCK INJECTION, Daiichi Sankyo, Inc., Tokyo, Japan; 0.4 mg/kg/h) was used to prevent electrical contamination of muscular activity. At the end of the experiment, we injected a ganglionic blocker, hexamethonium bromide (Sigma‐Aldrich; 60 mg/kg, bolus), to measure the residual noise level. During the experiments, we amplified the SNA signal using an amplifier (MEG‐6108; Nihon Kohden, Tokyo, Japan) and simultaneously displayed the signal on the computer screen, together with other hemodynamic parameters.

For volume loading, we collected blood from donor rats (*n* = 20). First, the rats were anesthetized and mechanically ventilated, as described before. Then, an 18‐gauge catheter was inserted from the right carotid artery and placed on the ascending aorta for blood sampling. After 60 min of stabilization, heparin sodium (HEPARIN SODIUM INJECTION 10000UNITS/10 mL, Nipro Co., Ltd, Osaka, Japan; 200 units/kg) with intravenous infusion (200 units/kg) was administrated to prevent coagulation, and blood was withdrawn for 1–2 min. The withdrawn blood was maintained at 37°C in a thermostatic chamber.

#### LPB activation under the ABR open‐loop condition

LPB was evoked by volume loading with the donated blood. The infusion dose was adjusted in each protocol. To assess the effect of LPB on SNA and AP under a wide range of CVP, we drained 6–9 mL/kg of blood to adjust AP at approximately 90 mmHg before conducting each protocol, and defined SNA and hemodynamics at the minimum CVP level as baseline. We opened the ABR loop by vascularly isolating bilateral carotid sinuses using previously established method (Saku et al. 2014, 2017; Sato et al. 1999). Briefly, we sectioned bilateral aortic depressor nerves to eliminate signals from baroreceptors in the aortic arch and subclavian arteries, and isolated bilateral carotid sinus arteries from the systemic circulation. We filled the isolated carotid sinuses with saline and controlled carotid sinus pressure (CSP) using a servo‐controlled piston pump (ET‐126A, Labworks Inc., CA).

### Protocols

#### Protocol 1: isolated effect of LPB on SNA

We fixed CSP at 90 mmHg to abolish buffering effects of ABR. We infused blood stepwise (3 mL/kg, 1 min/infusion) for 8 times to activate LPB. We compared changes in CVP, SNA, AP and HR from baseline with or without vagotomy.

#### Protocol 2: the effect of LPB on ABR function

We changed CSP stepwise from 60 to 160 mmHg every 20 sec and compared the responses of SNA, AP and HR at 3 levels of volume status: baseline (before volume loading), low volume loading (Low, 9 mL/kg), and high volume loading (High, 24 mL/kg).

### Data analysis

We digitized recorded signals at 200 Hz using a 16‐bit analog‐to‐digital converter (PowerLab 16/35; ADInstruments, NSW, Australia) and stored in a laboratory computer system. We analyzed SNA and AP averaged for the last 10 sec of each step. We evaluated SNA changes in each rat. In protocol 1, the measured SNA was normalized by the baseline SNA (100%). In protocol 2, the measured SNA was normalized by the baseline SNA obtained when the CSP equaled 60 mmHg (100%). We calculated the parameters of total (CSP‐AP relationship), central, and peripheral arcs using 6‐point data obtained from individual rats in protocol 2. Curve (ABR total and central arcs) and linear (ABR peripheral arc) regressions were conducted using the least‐squares method and Microsoft Excel (Excel 2013, Microsoft Japan Co., Ltd., Tokyo, Japan).

In the ABR open‐loop condition, ABR total and central arcs approximate an inverse sigmoid curve, and are quantified using a four‐parameter logistic function as follows (Kent et al. 1972):

$$y=\frac{P_{1}}{1+\exp\left[P_{2}\left(x-P_{3}\right)\right]}+P_{4}$$

where *x* and *y* represent the input (CSP) and the output (SNA or AP), respectively; *P*_*1*_ is the response range of y; *P*_*2*_ is the slope coefficient; *P*_*3*_ is the midpoint of the sigmoid curve on the x axis and *P*_*4*_ is the minimum value of y. We estimated the maximum gain (*G*_max_) with the formula **−**(*P*_*1 *_× *P*_*2*_)/4 at *x* = *P*_*3*_. The SNA–AP relation was defined as the ABR peripheral arc. This relation approximates a straight line, and is quantified using a linear regression as follows (Saku et al. 2014, 2017):

y=a×x+b

where *x* and *y* represent the input (SNA) and the output (AP), and *a* and *b* indicate the slope and the y‐intercept, respectively.

We determined the AP and SNA values at the operating point in each rat by the intersection between the ABR central and peripheral arcs. AP_OP_ and SNA_OP_ were represented as follows:

$${\mathrm{SNA}}_{\mathrm{OP}}=\frac{P_{1}}{1+\;\exp[P_{2}\left(AP_{\mathrm{OP}}\;-\;P_{3}\right)]}+P_{4}$$

$$AP_{\mathrm{OP}}=\;a\times \;\mathrm{SN}A_{\mathrm{OP}}+b$$

where *P*_1_, *P*_2_, *P*_3_, *P*_4_, a and b are constants identified from each rat in protocol 2.

### Statistical analysis

Data are expressed as means ± SEM. We compared the volume loading‐induced changes in SNA and AP from baseline using one‐way ANOVA with post‐hoc Dunnett test. The parameters of the ABR central and peripheral arcs were compared among the baseline, low, and high values using one‐way analysis of variance (ANOVA) with a post hoc Tukey test. The coefficient of determination (*R* ^2^) was also calculated for each individual as an index of fitting accuracy for ABR total, central, and peripheral arcs. Differences were considered significant when *P *<* *0.05.

## Results

### The effect of LPB on SNA in ABR open loop condition

**Figure 1:**
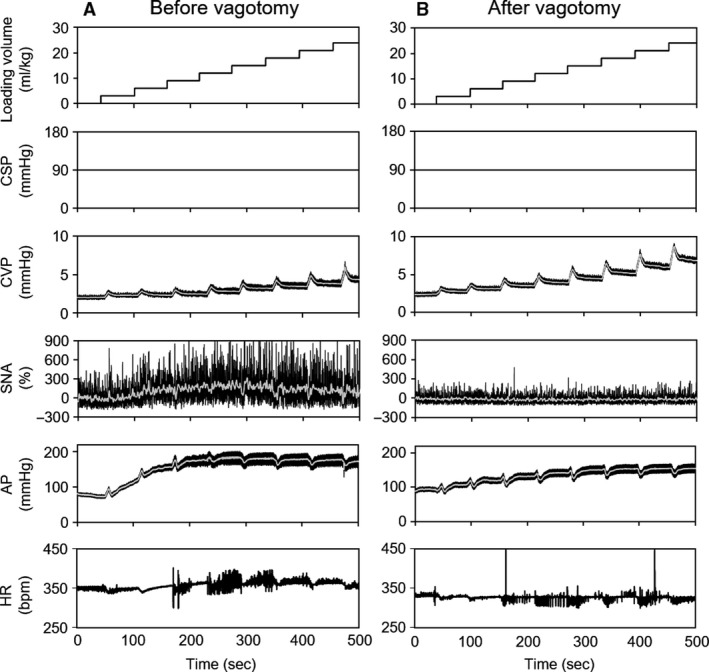
Time series data of CSP, CVP, SNA, AP, and HR in response to stepwise volume loading. The left and right panels present data before (A) and after (B) vagotomy, respectively. CSP, carotid sinus pressure; CVP, central venous pressure; SNA, % change in sympathetic nerve activity from baseline; AP, arterial pressure; HR, heart rate.

Figure 1 shows typical time series of CSP, CVP, SNA, AP, and HR under stepwise volume loading. CSP was maintained at 90 mmHg to abolish ABR. Volume loading increased CVP in a stepwise manner. In contrast, SNA increased during initial volume loading and declined with further volume loading. Bilateral vagotomy totally abolished the changes in SNA.

**Figure 2:**
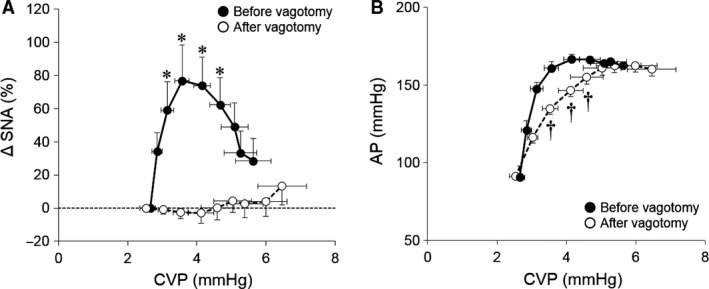
Responses of SNA (A) and AP (B) to changes in CVP. Solid line with closed circle (●) and dashed line with open circle (○) represent the data before and after vagotomy, respectively. Volume loading initially increased SNA with the maximum elevation of 76.8 ± 21.6% from baseline, whereas further volume loading significantly decreased SNA. Bilateral vagotomy totally abolished the changes in SNA in response to volume loading. CVP, central venous pressure; ΔSNA, % change in sympathetic nerve activity from baseline; AP, arterial pressure. * *P *<* *0.05, vs. baseline SNA, ^†^ *P *<* *0.05, vs. before vagotomy AP under the same volume loading condition.

As summarized in the left panel of Figure 2, the volume loading at CVP of 3.6 ± 0.2 mmHg significantly increased SNA with the maximum elevation of 76.8 ± 21.6% from baseline, whereas further volume loading significantly decreased SNA toward the baseline level. Bilateral vagotomy totally abolished the changes in SNA in response to volume loading. In terms of AP response, stepwise volume loading markedly elevated AP, while further volume loading above CSP of 4 mmHg had no further effect on AP. Bilateral vagotomy attenuated the increment of AP in response to low volume loading but had no effect on high volume loading. These changes in SNA and AP indicated that volume loading activated LPB and altered SNA in a two‐phase manner (an initial increase followed by decline) via vagal afferent nerves.

### The effect of LPB on ABR function

**Figure 3:**
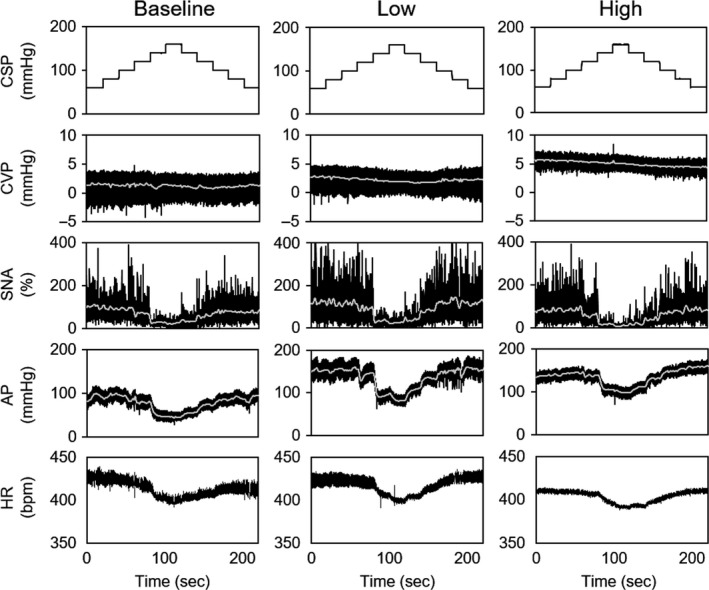
Time series data of CSP, CVP, SNA, AP, and HR at baseline (A), low (B), and high (C) volume loading conditions. CSP was changed stepwise from 60 to 160 mmHg and then back to 60 mmHg with step size of 20 mmHg. SNA measured was normalized by baseline SNA at CSP = 60 mmHg (100%). CSP, carotid sinus pressure; CVP, central venous pressure; SNA, sympathetic nerve activity; AP, arterial pressure. HR, heart rate.

Figure 3 presents typical time series of CVP, SNA, AP, and HR in response to stepwise changes in the CSP at baseline (Fig. 3A), low (Fig. 3B), and high (Fig. 3C) volume loading conditions. Volume loading significantly increased CVP (baseline: 2.7 ± 0.2, Low: 3.6 ± 0.4 and High: 5.4 ± 0.4 mmHg, *P *<* *0.05). Under all three conditions, changes in the CSP, in turn, changed SNA, AP, and HR reciprocally. Low volume loading increased SNA and AP compared to baseline, while high volume loading decreased SNA compared to low volume loading.

**Figure 4:**
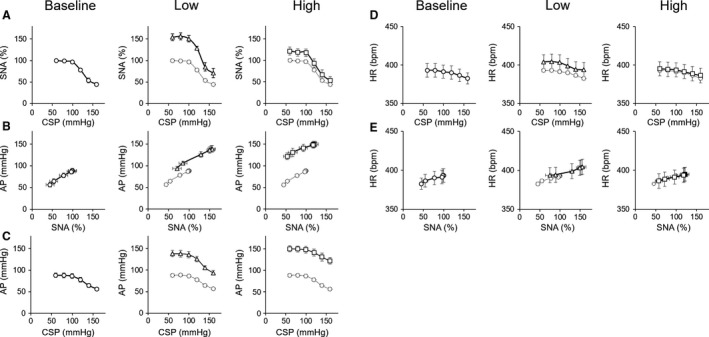
The open‐loop characteristics of the arterial baroreflex central (CSP‐SNA relationship), peripheral (SNA‐AP relationship) and total (CSP‐AP relationship) arcs under baseline (A), low (Low, B) and high (High, C) volume loading conditions. CSP‐HR (D) and SNA‐HR (E) relationships are also shown. Dotted lines in figures of Low and High are transcribed from baseline arterial baroreflex characteristics. CSP, carotid sinus pressure; SNA, sympathetic nerve activity; AP, arterial pressure; HR, heart rate; Low, low volume loading; High, high volume loading

Figure 4 shows the effects of volume loading‐induced LPB on ABR central, peripheral, and total arcs. Low volume loading shifted the ABR central arc upward compared to baseline (Fig. 4B), while high volume loading shifted back the ABR central arc toward baseline (Fig. 4C). The peripheral and total arcs were shifted upward as volume loading increased. Figures 4D and E show CSP–HR and SNA–HR relationships, respectively. The general trends in HR followed the changes in SNA.

Table 1 summarizes the parameters of ABR central, peripheral, and total arcs, and AP and SNA at the operating point. Low volume loading significantly increased *P*_*4*_ of the ABR central arc compared to baseline, while high volume loading decreased *P*_*4*_ significantly compared to low volume loading. There were no significant changes in *P*_*1*_, *P*_*2*_, *P*_*3,*_ or *G*_max_ from baseline, irrespective of volume loading condition. These data indicated that volume‐loading activated LPB altered SNA through resetting the ABR central arc. In the ABR peripheral arc, volume loading significantly increased the *y*‐axis intercept without changing the slope. In the ABR total arc, low volume loading significantly increased *P*_*1*_ (which indicates AP response range), *P*_*4,*_ (which indicates minimum AP), and the maximum gain. However, high volume loading did not change these parameters compared to baseline. At the operating point, AP monotonically increased with volume loading, while SNA peaked at low volume loading and decreased with further volume loading.

**Table 1:** Parameters of arterial baroreflex function, and arterial pressure and sympathetic nerve activity at the operating point (intersection of ABR central and peripheral arcs)

Parameters of ABR central arc	Baseline	Low	High
* P*_1_ (%)	60.7 ± 5.7	82.3 ± 15.1	70.2 ± 13.0
* P*_2_ (%/mmHg)	0.11 ± 0.01	0.18 ± 0.06	0.16 ± 0.05
* P*_3_ (mmHg)	127 ± 5	127 ± 3	129 ± 5
* P*_4_ (%)	38.9 ± 5.7	72.2 ± 11.2*	52.8 ± 10.0†
* G*_max_ (%/mmHg)	−1.7 ± 0.2	−3.2 ± 0.7	−2.2 ± 0.3
* *Coefficient of determination (*R* ^2^)	0.981 ± 0.005	0.975 ± 0.010	0.963 ± 0.023

Parameters of ABR peripheral arc	Baseline	Low	High
a (mmHg/%)	0.59 ± 0.10	0.54 ± 0.09	0.47 ± 0.08
b (mmHg)	30 ± 7	58 ± 9*	98 ± 8*†
Coefficient of determination (*R* ^2^)	0.977 ± 0.006	0.963 ± 0.015	0.976 ± 0.006

Parameters of ABR total arc	Baseline	Low	High
*P*_1_ (mmHg)	34 ± 5	53 ± 7*	43 ± 9
*P*_2_ (mmHg/mmHg)	0.11 ± 0.02	0.11 ± 0.01	0.19 + 0.07
*P*_3_ (mmHg)	125 ± 4	128 ± 4	135 ± 7
*P*_4_ (mmHg)	55 ± 4	88 ± 6*	102 ± 9
*G*_max_ (mmHg/mmHg)	−0.9 ± 0.2	−1.6 ± 0.3*	−1.3 ± 0.3
Coefficient of determination (*R* ^2^)	0.997 ± 0.001	0.996 ± 0.002	0.988 ± 0.010

Operating point	Baseline	Low	High
AP_OP_ (mmHg)	88 ± 5	120 ± 4*	130 ± 8*†
SNA_OP_ (%)	97.6 ± 1.0	121.1 ± 7.7*	76.7 ± 7.1†

Summarized data of parameters of the arterial baroreflex (ABR) central, peripheral, and total arcs, and AP and SNA at the operating point (intersection of central and peripheral arcs). *P*_1_, response range of SNA or AP; *P*_2_, coefficient of gain; *P*_3_, midpoint of the operating range; *P*_4_, minimum SNA or AP; G_max_, maximum gain; a, slope; b, intercept; AP_OP_, AP at the operating point; SNA_OP_, SNA at the operating point.

*P <* *0.05, versus baseline, ^†^ *P *<* *0.05, versus Low.

**Figure 5:**
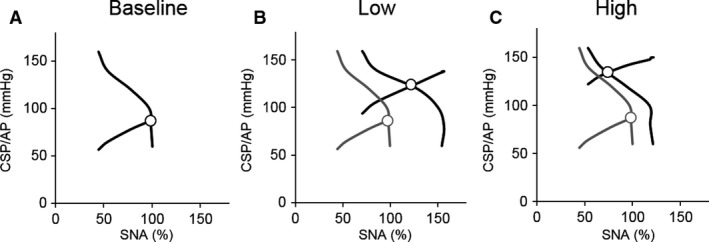
Arterial baroreflex (ABR) equilibrium diagrams under baseline (A), low (Low, B), and high (High, C) volume loading conditions. Each equilibrium diagram was obtained by superimposing the ABR central and peripheral arcs using averaged values in each parameter (Table 1). Open circles (○) represent the operating point. Gray lines in Low and High are transcribed from the baseline arterial baroreflex equilibrium diagram. CSP, carotid sinus pressure; SNA, sympathetic nerve activity; AP, arterial pressure.

Figure 5 shows the ABR equilibrium diagrams obtained by superimposing the ABR central and peripheral arcs using averaged values in each parameter. The operating point, the intersection of the ABR central and peripheral arcs, for each of the volume loading condition is depicted. Gray lines represent the equilibrium diagram transcribed from baseline for comparison. Low volume loading shifted the operating point upward and to the right compared to baseline (Fig. 5B). In contrast, high volume loading slightly shifted the operating point upward and to the left compared to low loading (Fig. 5C).

## Discussion

Major findings of the present study are as follows; (1) volume loading‐induced LPB evoked two‐phase changes, an initial increase followed by decline from the baseline value, in SNA under ABR open loop condition. (2) LPB altered SNA via resetting of the ABR central arc.

### The isolated impact of LPB activation on SNA and AP

Because the ABR is a powerful negative feedback system that stabilizes AP against exogenous disturbances, it may buffer LPB‐evoked changes of SNA and AP. Hence, it is not possible to capture the LPB function accurately under closed‐loop conditions. Thus, we opened the arterial baroreflex loop and examined the isolated impact of volume loading‐induced LPB activation on SNA and AP in this study. As shown in Figures. 1 and 2, stepwise volume loading within CVP < 4 mmHg increased SNA by 76.8 ± 21.6% from baseline. In contrast, further volume loading decreased SNA toward the baseline level. These findings indicate that volume loading activates LPB and alters SNA depending on the degree of volume status and volume loading.

Several investigators have reported the effects of LPB on SNA and hemodynamics. Nonidez (1937) documented the presence of receptors in the atrium and pulmonary vasculature similar to carotid baroreceptors. Carswell et al. (1970) reported that stimulation of left or right atrium with balloon inflation increased cardiac SNA and thus HR whereas did not increase SNA to the hind limbs. Karim et al. (1972) reported that activation of left atrial receptors by distension of balloons at the pulmonary vein‐atrial junctions increased cardiac SNA and HR, decreased renal SNA, while did not affect lumbar or splenic SNA. Paradoxical alteration in cardiac SNA by LPB was also reported previously. Hakumäki (1979) measured the hemodynamic responses to 42 intravenous volume infusions of saline in dogs, and demonstrated that 31 infusions increased cardiac SNA and HR, while 11 infusions decreased cardiac SNA and HR through sympatho‐inhibition and parasympathetic activation. In addition, Kollai et al. (1978) reported that left atrium stimulation increased cardiac SNA following immediate decrease of cardiac SNA. On the other hand, several studies have shown that the cardio‐pulmonary receptors sensed blood accumulation by volume loading and inhibited renal SNA (Ricksten and Thoren 1980; Morita and Vatner 1985; Badoer et al. 1998; Pyner et al. 2002). Previous experiments and our results suggested that LPB through baroreceptors in the cardiopulmonary region can substantially change SNA. The results also indicated that the SNA response against LPB activation varies among studies. We defined LPB as the baroreflex elicited by systemic volume infusion, and the LPB input as the central venous pressure (CVP). Since low‐pressure sensors are widely distributed in the cardiopulmonary region, our observation of LPB in this study revealed an integrated SNA response against systemic volume perturbation. This means that even if a few low‐pressure receptors in the cardiopulmonary region evoke a reflex in a different direction, we can observe only the net effect of LPB on the SNA response. The differences in the SNA recording site might also affect these results. Several afferent input signals are integrated in the brain stem of the nucleus of the solitary tract (Mifflin and Felder 2001), while the efferent output signals driven from the rostral ventrolateral medulla vary among projected organs (Floras et al. 2001; Morrison 2001). Therefore, the amount and direction of local SNA signals can vary among renal SNA, cardiac SNA, and splanchnic SNA. In this study, our aim was to assess the impact of LPB activation on baroreflex‐controlled SNA; therefore, we recorded splanchnic SNA. Our previous studies suggested that baroreflex perturbation significantly changes splanchnic SNA with high coherence (Saku et al. 2014, 2017), indicating that measuring splanchnic SNA is enough for explaining central regulation of SNA. In addition, if we cut the efferent fibers of splanchnic nerve, we will be unable to assess the pressure response, because splanchnic SNA regulates the abdominal vascular bed, which mainly regulates blood pressure (Chaudhuri et al. 1992; Rowell et al. 1972). Further studies are needed to clarify how specific receptors in the cardiopulmonary region (e.g., the right atrium, left atrium and pulmonary vein) affect each local SNA. Our results suggest that whether LPB excites or inhibit splanchnic SNA depends on the degree of volume status and volume loading.

We also showed that bilateral vagotomy totally abolished the changes in SNA, confirming that the vagal nerves are involved in the afferent pathway of LPB. Thus, the vagal nerves transmit signals, not only from abdominal organs but also of intravascular volume status to the central nervous system, and play a pivotal role in circulatory homeostasis through autonomic modulation.

### Interaction between low pressure baroreflex and arterial baroreflex

The signals from various afferent inputs are known to converge in the nucleus tractus solitarius in the brainstem through afferent neural pathways and determine the level of SNA controlled by rostral ventral medulla. In this study, we elucidated how volume loading‐induced LPB interacts with ABR to affect the sympathetic nervous system. As shown in Figure 4, LPB induces resetting the central arc of ABR toward both upward and downward, but does not change the slope of the peripheral arc regardless of the magnitude of volume loading. It indicates that LPB is capable of altering the target AP (command pressure) of the ABR system (Sato et al. 1999) depending on the degree of volume status and volume loading.

Kashihara et al. (2003) demonstrated that Bezold‐Jarisch reflex induced by a 5‐HT3 receptor agonist reduced SNA and the gain of ABR central arc. We also reported that the electrical stimulation of afferent vagal nerve caused an almost parallel resetting of the ABR central arc and reduction of SNA (Saku et al. 2014). Regarding the interaction between vagal nerve and ABR as the afferent input, our results are partially consistent with these previous reports. However, increased SNA as a response to LPB stimulation shown in the present study has not been hitherto documented. We suspect that high‐intensity chemical or electrical stimulation activates the vagal nerves resulting in a dominant response of sympatho‐inhibition. Further investigation is needed to examine the effect of low dose of such stimulation on SNA.

### Physiological role of LPB in circulatory homeostasis

We observed that through ABR central arc resetting, volume loading–induced acute LPB activation evokes two‐phase changes in SNA: an initial increase followed by a decline from the baseline.

**Figure 6:**
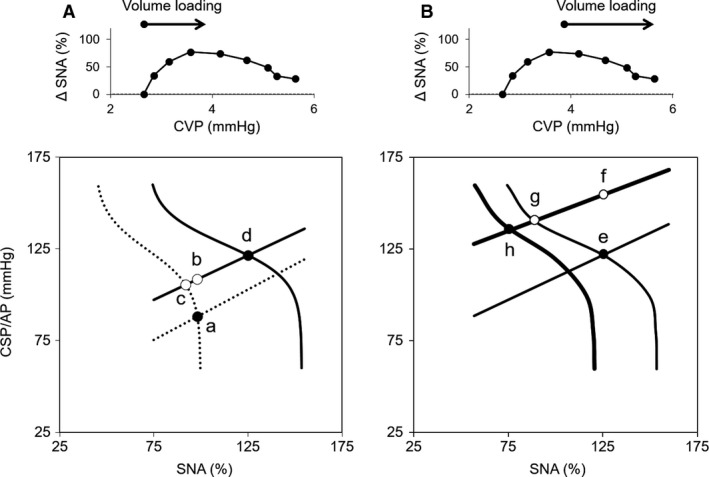
Equilibrium diagrams of arterial baroreflex (ABR) in the absence or presence of low pressure baroreflex (LPB) with low (A) and high (B) volume loading. Changes in the operating point in response to volume loading are shown. The upper and lower figures are adopted from Figure 2 and Figure 5, respectively. At low volume loading, the ABR peripheral arc shifts upward (dashed straight line to thin straight line) and elevates AP from a to b if ABR and LPB do not operate. The operating point moves to c and d in the presence of ABR and ABR with LPB, respectively. At high volume loading, the ABR peripheral arc further shifts upward (thin straight line to thick straight line) and elevates AP from e to f if ABR and LPB do not operate. The operating point moves to g and h in the presence of ABR and ABR with LPB, respectively. Closed (●) and open (○) circles indicate real and imaginary operating points, respectively. CSP, carotid sinus pressure; AP, arterial pressure; SNA, sympathetic nerve activity.

In the initial volume‐loading phase, we observed an increase in SNA through upward shifting of the ABR central arc against volume loading, which did not make sense in terms of AP regulation. Figure 6A is a scheme adopted from Figure 5B. Figure 6A graphically simulates the impact of LPB on ABR‐controlled AP regulation if the subject operates within a low‐CVP range. Assume the ABR central and peripheral arcs are in equilibrium (Fig. 6A, point a). Volume loading increases AP without changing SNA when LPB and ABR do not operate (Fig. 6A, point b). In contrast, the presence of ABR inhibits SNA and decreases AP (Fig. 6A, point c). However, the presence of LPB increases SNA and AP (Fig. 6A, point d). In this case, LPB opposes AP‐buffering effect of ABR. Conversely, volume unloading within the CVP range possibly suppresses SNA and further lowers AP. This might result in a catastrophic vicious cycle moving toward hemodynamic collapse. Therefore, we speculate that the sympatho‐excitatory response in the initial volume‐loading phase might represent unusual reactions known as Bainbridge reflex (Bainbridge 1915; Bergström et al. 1971) and Bezold–Jarisch reflex (Bell et al. 1993; Mark 1983), which are observed during hemorrhage or hypovolemia.

On the other hand, the sympatho‐inhibitory response against volume loading might be reasonable from the point of view of AP regulation. Figure 6B graphically simulates the effect of LPB on ABR‐controlled AP regulation if the subject operates within a high‐CVP range. Assume the ABR central and peripheral arcs are in equilibrium (Fig. 6B, point e). Volume loading increases AP without changing SNA when LPB and ABR do not operate (Fig. 6B, point f). In contrast, the presence of ABR inhibits SNA and decreases AP (Fig. 6B, point g), and the presence of both LPB and ABR further inhibits SNA and decreases AP (Fig. 6B, point h). These data indicate that in response to volume loading, LPB augments AP stability in cooperation with ABR, that is, LPB makes the volume loading of the circulatory system tolerant. In this study, we opened the chest to minimize the effect of intrathoracic pressure (=0 mmHg) on hemodynamics. However, LPB senses transmural pressure, so a change in intrathoracic pressure might, in turn, change the LPB response. Since intrathoracic pressure is negative with spontaneous breathing (Angell James 1971), the CVP–SNA relationship (Fig. 2) may shift leftward under physiological conditions. This makes LPB in the physiological operating range sympatho‐inhibitory in response to volume loading. Further studies are needed to examine how intrathoracic pressure with spontaneous breathing alters the SNA response against volume loading.

### Limitations

There are several limitations in this study. First, we conducted this study under anesthetic condition. Since anesthesia is known to alter the autonomic and cardio‐vascular functions, we cannot extrapolate these results directly to physiological and conscious conditions.

Second, as discussed above, the effect of intrathoracic pressure has to be considered. Thus, the effects of LPB on SNA and hemodynamics under conscious condition should be studied to interpret the physiological impact of LPB.

Third, we examined, in this study, the acute effects of LPB on SNA and ABR function under ABR open loop condition. However, previous studies have suggested that LPB regulates not only SNA but also hormonal factors such as the release of renin, vasopressin and atrial natriuretic peptide (ANP) (Carr et al. 1992; Grassi et al. 1988), and the impairment of LPB induced sympatho‐inhibitory effect worsens the severity of hypertension (Mancia et al. 1988). Further investigations are needed to understand the role of LPB on long term as well as short term circulatory homeostasis.

## Conclusions

Volume loading‐induced LPB evoked two‐phase changes in SNA via upward or downward resetting of the ABR central arc. LPB may contribute greatly to stabilize AP in response to volume status.

## Conflict of Interest

Oga Y, Nishikawa T, Tobushi T, Hosokawa K, Tohyama T and Sakamoto T have nothing to declare. Saku K and Kishi T work in a department endowed by Omron Healthcare Co. Sunagawa K works in a department endowed by Omron Healthcare Co. and Actelion Pharmaceuticals Japan. Tsutsui H received honoraria from Daiichi Sankyo, Inc., Otsuka Pharmaceutical Co., Ltd., Takeda Pharmaceutical Company Limited, Mitsubishi Tanabe Pharma Corporation, Boehringer Ingelheim Japan, Inc., Novartis Pharma K.K., Bayer Yakuhin, Ltd., Bristol‐Myers Squibb KK, and Astellas Pharma Inc., and research funding from Actelion Pharmaceuticals Japan, Daiichi Sankyo, Inc., and Astellas Pharma Inc.
